# The association between tobacco smoking and systolic toe pressures in active foot ulceration

**DOI:** 10.1038/s41598-024-59158-5

**Published:** 2024-04-12

**Authors:** Nada Bechara, Tien-Ming Hng, Jenny E. Gunton

**Affiliations:** 1 Centre for Diabetes, Obesity and Endocrinology (CDOE) Research, The Westmead Institute for Medical Research, Westmead, NSW 2145 Australia; 2 Department of Diabetes and Endocrinology, Blacktown Mt Druitt Hospital, Blacktown, NSW 2148 Australia; 3 School of Medicine, Western Sydney University, Blacktown Mt Druitt Hospital, Blacktown, NSW 2148 Australia; 4 Faculty of Medicine and Health, Westmead Hospital, Sydney Medical School, The University of Sydney, Westmead, NSW 2145 Australia

**Keywords:** Toe pressures, Foot ulcer, Ischaemia, Ulcer healing, Lower limb amputation, Smoking, Outcomes research, Diagnosis, Risk factors, Comorbidities

## Abstract

Smoking may increase the risk of diabetic foot disease and ulceration. It does so by impairing glycaemic control and promoting the formation of advanced glycated end-products. Additionally, smoking is known to delay surgical wound healing and accelerate peripheral arterial disease. We aimed to determine whether toe pressures differed in smokers with a foot ulcer, when compared to non-smokers and ex-smokers, as well as ulcer outcomes at 12 months, among patients attending Blacktown Hospital High Risk Foot Service (HRFS). This study is a retrospective analysis of our prospectively collected clinic database. Eligible participants were adults attending the HRFS between June 2020 and April 2022. Participants were included if they had an ulcer, at least one systolic toe pressure reading completed at their initial visit and attended at least one follow-up visit. Participants were followed until healing, loss to follow-up or a minimum of 12 months. A total of 195 participants were included; 36 smokers, 82 ex-smokers, and 77 controls who had never smoked. Smoking status was by self-report. Current smokers were significantly younger at initial presentation (*p* = .002) and tended towards lower socioeconomic status (*p* = .067). Current smokers were significantly more likely to have ischaemic grade 3 toe pressures (< 30 mmHg) of their left foot (*p* = .027), suggestive of reduced perfusion. At the end of follow up period, smokers had the numerically highest rates of minor amputations. In conclusion, smokers ulcerate younger and are more likely to have grade 3 ischaemia. Collecting information about the brachial artery pressures and the time since the last cigarette may clarify any relationship between smoking and toe pressures.

*Trial registration*: WSLHD HREC ethics approval 2111-02 and ANZCTR registration 382470. Registered on 15/09/2021.

## Introduction

**Figure 1:**
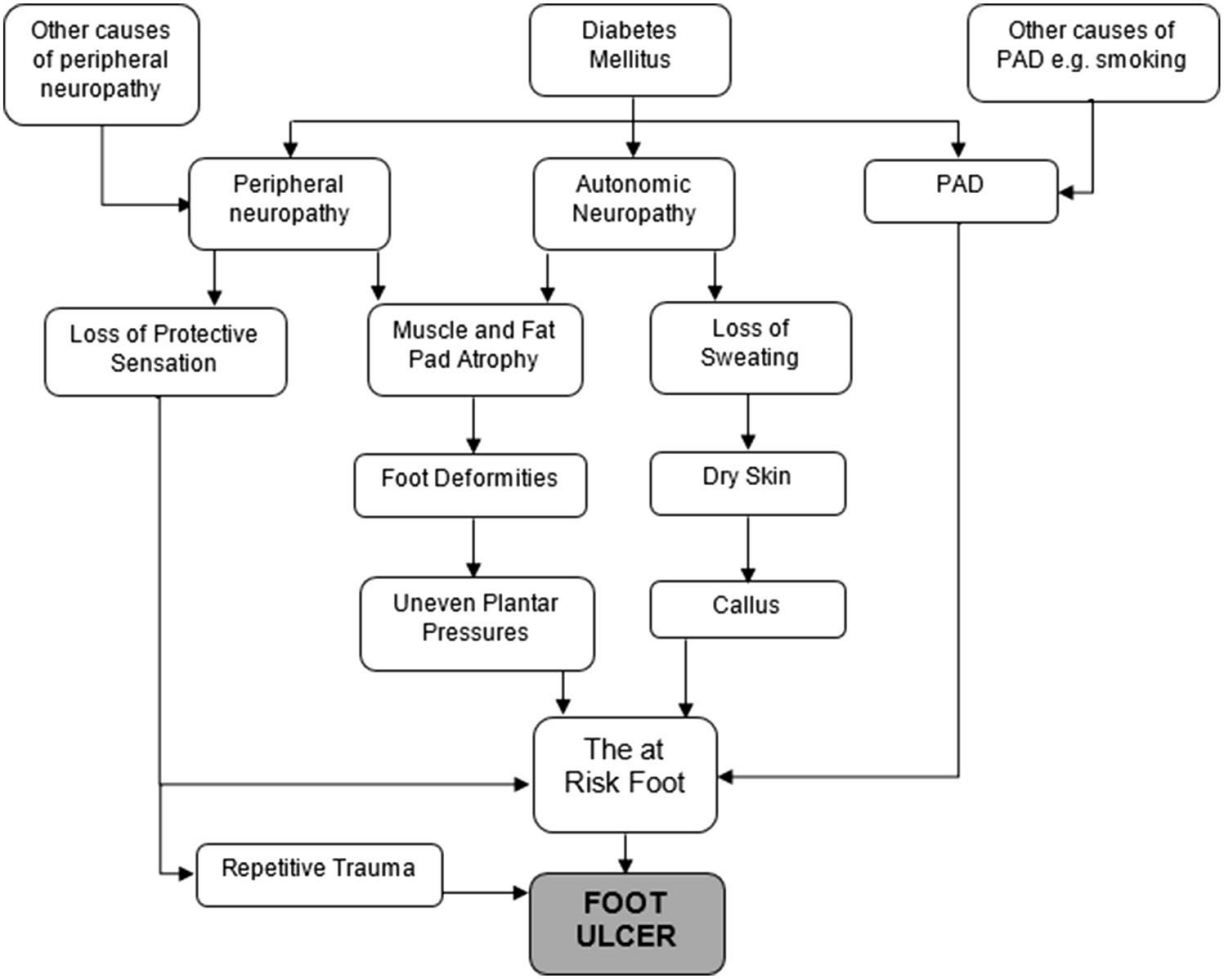
Potential pathways to foot ulceration.

Foot ulceration is an important cause of hospitalisation and lower limb amputation (LLA) worldwide ^1^. Most foot ulcers have a multifactorial pathophysiology (Fig. 1). Peripheral neuropathy, peripheral arterial disease (PAD), foot deformity, poor glycaemic control, tobacco smoking, duration of diabetes and the presence of other micro and macro vascular complications all contribute to the pathogenesis ^2–4^. The majority of non-traumatic LLA are preventable; hence it is important to identify factors contributing to ulceration, delayed healing and LLA. LLA encompasses major or minor foot amputation, such as digital, foot, below knee, or above knee amputation.

Tobacco smoking is decreasing worldwide but remains a serious problem ^5^. According to the National Drug Strategy Household Survey (NDSHS) in 2019, it was estimated that 11.2% of Australian adults smoked daily ^6^. These rates are unfortunately not lower in people with diabetes, with the most recent Australian National Diabetes Audit report finding 495 of 4334 people currently smoked (11.4%) ^7^. Smoking is associated with many chronic illnesses including cardiovascular disease, cancer, diabetes and lung disease ^8^. Interestingly, smoking increases the risk of developing diabetes ^9^. Current cigarette smoking is associated with deterioration in glucose control, as measured by HbA1c ^10,11^.

PAD is narrowing of the arteries in the limbs due to atherosclerosis and is much more common in the lower limb vessels ^12^. PAD which impairs blood flow, which impairs wound healing, and increases the risk of infection. Patients with PAD are more likely to develop critical limb ischaemia (CLI), which may be diagnosed in the presence of ischaemic foot ulcers, gangrene or ischaemic rest-pain ^13^.

Smoking is closely linked to the formation of vascular plaques and previous studies have shown strong associations between smoking and PAD and cardiovascular disease ^14,15^. Chronic smokers have weakened vasodilation in the microvasculature which can also further decrease the already reduced blood flow due to PAD ^8,16^. It is reported that amputation rates related to PAD in smokers is twice the rate of that in non-smokers ^17^.

Smoking is associated with higher risks of postoperative complications, including delayed healing of surgical wounds and increased risk of wound infection ^18,19^. Studies have reported that smoking cessation for at least 3 weeks prior to surgery reduces postoperative morbidity from 52 to 18% ^8^. Thus, smoking appears likely to be important in foot wound healing as well.

In past years, the ankle brachial index (ABI) has been the preferred method in assessing for lower limb PAD. However, ABI has been found to lack accuracy for those with diabetes who may have medial arterial calcification – a condition of medial arterial stiffness that falsely elevates the ABI ^20,21^. Therefore, toe brachial index (TBI) or systolic toe pressures is the recommended screening method of choice in high risk foot services nation-wide ^22^. Systolic toe pressures and TBI have been shown to have great inter- and intra-clinician reliability in people with and without diabetes ^23^.

In clinical settings, toe pressure is measured using photoplethysmography (PPG). PPG assesses blood flow by emitting an infrared light that is reflected by the red blood cells in superficial vessels. The amount of reflected light corresponds to pulsatile changes and tissue blood volume ^24^. Toe PPG provides a functional assessment of perfusion status. It is widely accepted that a toe pressure reading of ≥ 60 mmHg is considered normal ^25,26^. A toe pressure < 30 mmHg is the cut off for CLI where patients may begin to experience rest pain, and is considered to be grade 3 ischaemia ^26^.

Our primary outcome was to assess toe pressures amongst current smokers and compare these to the non-smoking and ex-smoking cohorts. Our secondary outcomes were amputation rates at 12 months, ulcer healing at 12 months, age at ulceration, diabetes prevalence, diabetes complications, and socioeconomic status (SES) amongst the smoking cohort compared to ex and non-smokers.

## Methods

Ethical approval was granted by Western Sydney Local Health District (WSLHD) Human Research Ethics Committee (Application Number 2111–02) prior to data extraction. This study was performed in accordance with the ethical standards as laid down in the Declaration of Helsinki. This ANZCTR registered study was a retrospective analysis of our prospectively collected clinic database. Demographic and ulcer data was collected at each clinic visit and entered into the Microsoft Access database (Microsoft Corp., Washington, USA). Eligible participants were any adult attending Blacktown Mount Druitt Hospital High Risk Foot Service (HRFS) with a new ulcer between June 2020 and April 2022. Participants were included if they had at least one systolic toe pressure reading taken at their initial presentation and at least one follow-up visit to the service to be able to assess for ulcer outcome. Participants were followed until healing, loss to follow-up or for at least one year from baseline visit to assess for outcomes. Participants were classified as 'not healed' if they were not healed by 12 months from their initial visit, or if they were not healed at the time of loss to follow-up. Participation was open to patients both with and without diabetes.

Patient data was extracted from our clinic database. This included age, sex, medical history, medications, diabetes status, year of diabetes diagnosis, history of vascular surgery, macrovascular complications, and microvascular complications. Smoking status, previous ulceration, and site of recurrent ulceration was self-reported.

Neurological status was determined as a loss of protective sensation (LOPS) using two testing methods, monofilament, and vibration perception, with failure of either test positive for LOPS. A Semmes–Weinstein (North Coast Medical, California, USA) 10-g monofilament was tested at ten sites of each foot, with failure to detect 4 or more locations considered neuropathic ^27^. Vibration perception was tested using a neurosthesiometer (Wilford Industrial, Nottingham, UK) with neuropathy classified at unable to detect > 25 V ^28^.

### Toe pressure assessment

Systolic toe pressures were measured using the Smartdop 30EX (Hadeco Inc., Kawasaki, Japan) which is an automated photoplethysmography (PPG) unit. Initially a 2.5 cm x 9 cm digital cuff was placed on the proximal aspect of the hallux and the PPG probe was secured onto the pulp of the hallux with hypoallergenic tape. A 1.9 cm cuff was used for patients with smaller toes or if taking the measurement from the 2^nd^ digit. The reading is measured after the patient has been resting supine for at least 15 min. Once the machine detects a waveform it will signal that it is ready to calculate a reading and the inflate button can be pressed to calculate a systolic toe pressure. Systolic toe pressure readings were taken at both feet. The halluces are the preferred site for measurement, however if there had been a previous hallux amputation the measurement can also be completed on the 2^nd^ digit with good reliability ^27^. Where 1^st^ and 2^nd^ digits had been previously amputated, no pressure recording was made for that side. If a participant only had a reading for one foot, they were included. People with no toe pressure readings were excluded.

### Anthropometric measurements

Height and weight measurements were extracted from medical records. These measurements were taken at the patients most recent admission to Blacktown or Westmead Hospital using a SECA electronic scale and stadiometer, by a nurse.

### Ulcer measurements

Ulcer measurements are calculated at each clinic visit which ranges anywhere from 1–4 weeks apart. An image of the ulcer is captured using the SilhouetteStar™ (ARANZ medical, Christchurch, NZ) which is a three-dimensional wound camera that gives the length, width, depth, and area of the ulcer. This specific type of camera has been shown to have good reliability and validity ^28,29^.

### Socioeconomic status (SES)

Socioeconomic status was taken from the Australian Bureau of Statistics Index of Relative Socio-economic Disadvantage (IRSD) map, accessed at 2033.0.55.001—Census of Population and Housing: Socio-Economic Indexes for Areas (SEIFA), Australia, 2016 (abs.gov.au). The postcode is populated into the interactive map and an IRSD number between 1 and 5 is given, with 1 being the most disadvantaged.

### Statistical analysis

Data was analysed using the Statistical Package Social Science software version 22.0 (SPSS Science, Chicago, Illinois, USA). Smokers, ex-smokers, and non-smokers were used as the main groups for statistical analysis. Toe pressures of these groups were compared as well as their ulcer measurements at each visit and final ulcer outcomes (healed, lost to follow up, or amputated) at 12 months using one-way ANOVA. All other categorical dependent variables were compared using one-way ANOVA. The Kruskal–Wallis Test was used to determine statistical significance of the results, with a p value ≤ 0.05 deemed statistically significant.

*Ethical approval.*

Consent to participate was obtained from Western Sydney Local Health District (WSLHD) Human Research Ethics Committee (Application Number 2111–02). Informed consent was obtained from all subjects and/or their legal guardian(s).

## Results

One hundred and ninety-five participants were included in the study. Of these, 36 were current smokers, 82 were ex-smokers and 77 had never smoked (Table 1). All participants had at least one toe pressure reading measured to be included in the study. There were no missing toe pressures of left feet due to previous amputations, and 14 missing values for right feet. If no signal was detected on the PPG machine, due to PAD, this was entered as a toe pressure of 0 mmHg for statistical analysis purposes (Table 2).

**Table 1:** Participant demographic and medical characteristics.

	Whole group	Current smokers	Ex-smokers	Non-smokers	*P*
N (%)	195	36 (18.5)	82 (42.1)	77 (39.5)	
Male / females, n (%)	134 / 61	26 (72.2) / 10 (27.8)	63 (76.8) / 19 (23.2)	45 (58.4) / 32 (41.6)	**.044**
Age, median years (IQR)	66.7 (55.9–74.9)	59.5 (47.7–70.1)	69.9 (59.4–77.3)	66.0 (56.6–75.4)	**.002**
Body mass index, median kg/m2 (IQR)	31.2 (27.1–36.1)	29.8 (24.8–34.5)	32.8 (27.7–36.5)	32.4 (27.2–37.2)	.537
Weight, median kg (IQR)	93 (76.1–112)	88 (75–107)	98 (80–112)	93 (75–112.9)	.836
Height, median cm (IQR)	173 (162–180)	175 (165–182)	170 (164–179.5)	171 (160–180)	.997
IRSD, n (%)					.067
1 – most disadvantaged	52 (26.7)	12 (33.3)	21 (25.6)	19 (24.7)	
2	100 (51.3)	20 (55.6)	44 (53.7)	36 (46.8)	
3	23 (11.8)	1 (2.8)	13 (15.9)	9 (11.7)	
4	5 (2.6)	0	3 (3.7)	2 (2.6)	
5 – most advantaged	15 (7.7)	3 (8.3)	1 (1.2)	11 (14.3)	
Diabetes, n (%)	169 (86.7)	29 (80.6)	78 (95.1)	62 (80.5)	**.010**
T1D, n (%)	9 (4.6)	3 (8.3)	5 (6.1)	2 (2.6)	
T2D, n (%)	160 (82.1)	26 (72.2)	73 (89)	60 (77.9)	
No diabetes, n (%)	26 (13.3)	7 (19.4)	4 (4.9)	15 (19.5)	
Peripheral arterial disease, n (%)	64 (32.8)	8 (22.2)	32 (39)	24 (31.2)	.182
Ischaemic heart disease, n (%)	47 (24.1)	6 (16.7)	23 (28.1)	18 (23.4)	.417
Cerebrovascular disease, n (%)	8 (4.1)	2 (5.6)	4 (4.9)	2 (2.6)	.707
Peripheral neuropathy, n (%)	171 (87.7)	30 (83.3)	79 (96.3)	62 (80.5)	**.009**
Retinopathy, n (%)	55 (28.2)	8 (22.2)	29 (35.4)	18 (23.4)	.191
Nephropathy, n (%)	46 (23.6)	5 (13.9)	25 (30.5)	16 (20.8)	.122
Previous LLA, n (%)	70 (35.9)	10 (2.8)	28 (34.2)	32 (41.6)	.274
Previous ulcer, n (%)	106 (54.4)	14 (38.9)	48 (58.5)	44 (57.1)	.117
Previous revascularisation, n (%)	28 (14.4)	3 (8.3)	16 (19.5)	9 (11.7)	.210

*IRSD*  Index of relative socioeconomic disadvantage, *IQR* Interquartile range, *T1D* Type 1 diabetes, *T2D* Type 2 diabetes. Bolded values statistically significant with significance set at < 0.05.

**Table 2:** Results and Ulcer Outcomes.

	Whole Group	Current smokers	Ex-smokers	Non-smokers	*P*
N (%)	195	36 (18.5)	82 (42.1)	77 (39.5)	
Left toe pressures, median mmHg (IQR)	92 (57–122)	104 (66–125)	89 (56–124)	94 (54–121)	.665
Left toe pressures mmHg, n 178 (%)					
Ischaemia grade 0 (> 60)	144 (73.8)	28 (77.8)	59 (71.9)	57 (74.0)	.999
Ischaemia grade 1 (40–59)	15 (7.7)	0	8 (9.8)	7 (9.1)	.153
Ischaemia grade 2 (30–39)	6 (3.1)	0	2 (2.4)	4 (5.2)	.268
Ischaemia grade 3 (< 30)	13 (6.7)	6 (16.7)	5 (6.1)	2 (2.6)	**.027**
Right toe pressures, median mmHg (IQR)	98 (71–125)	97 (78–128)	93 (64–122)	106 (76–128)	.508
Right toe pressures mmHg, n 181 (%)					
Ischaemia grade 0 (> 60)	147 (75.4)	26 (72.2)	61 (74.4)	60 (77.9)	.379
Ischaemia grade 1 (40–59)	12 (6.2)	3 (8.3)	6 (7.3)	3 (3.9)	.613
Ischaemia grade 2 (30–39)	6 (3.1)	1 (2.8)	4 (4.9)	1 (1.3)	.383
Ischaemia grade 3 (< 30)	16 (8.2)	4 (11.1)	7 (8.5)	4 (5.2)	.556
Ulcer measurements					
Area baseline, median cm^2^ (IQR)	0.8 (0.3–3.1)	1.0 (0.2–3.5)	0.74 (0.3–3)	0.82 (0.4–3.2)	.336
Area follow up 1, median cm^2^ (IQR)	0.6 (0.1–2.3)	0.4 (0.08–2.8)	0.4 (0.1–2.6	0.61 (0.1–2.3)	.108
Ulcer outcome at 12 months, n 195					
Healed, n (%)	81 (41)	16 (44.4)	40 (48.8)	25 (32.5)	.215
Not healed/ongoing, n (%)	49 (25.6)	10 (27.8)	17 (20.7)	22 (28.6)	.587
Amputated, n (%)	9 (4.6)	3 (8.3)	4 (4.9)	2 (2.6)	.126
Lost to follow up, n (%)	56 (28.7)	7 (19.4)	21 (25.6)	28 (36.4)	.088

*IQR* Interquartile range. Bolded values statistically significant with significance set at < 0.05.

Of the 195 participants, 134 (68.7%) were male and 61 (31.3%) female (*p* = 0.044). This is consistent with the typical male to female ratio seen in a high risk foot service. The median age of all participants was 66.7 years and smokers were significantly younger than the whole group at 59.5 years (*p* = 0.002). Nine participants had type 1 diabetes, 160 had type 2 diabetes and 26 participants did not have diabetes.

**Figure 2:**
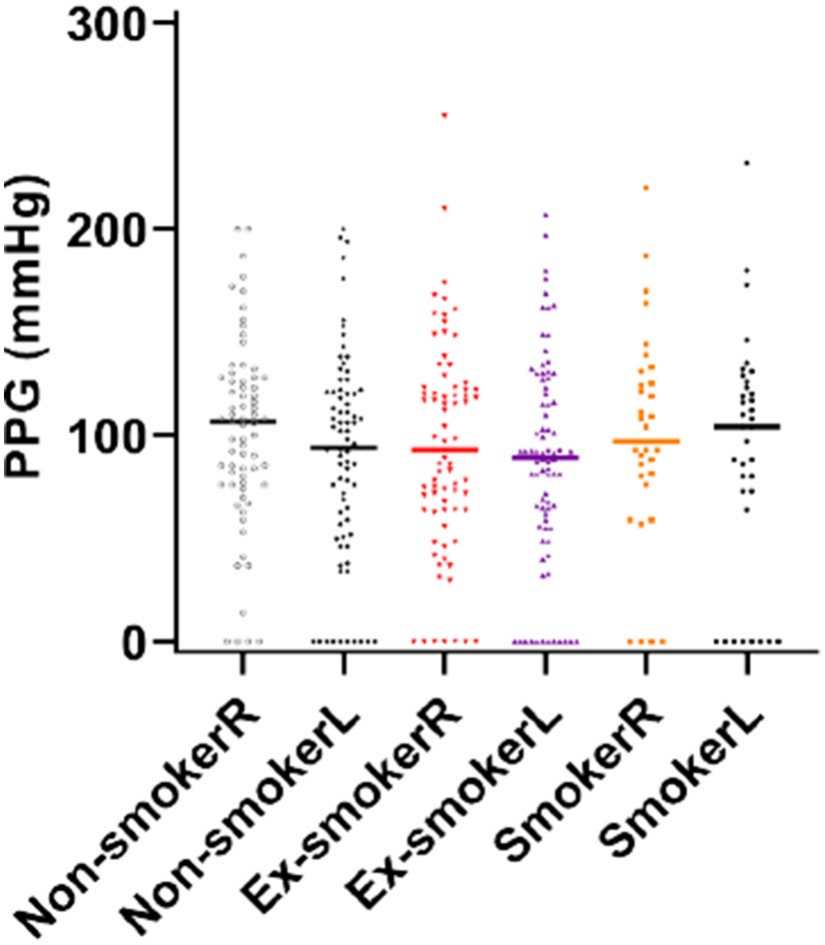
Photoplethysmography (PPG) results for left (L) and right (R) feet. Data shows individual results, and the lines indicate medians.

Ulcer size at baseline was largest in the smoking cohort, with greatest reductions at first follow up also noted in this group, though these results were not significant.The median toe pressure for the left foot was 92 mmHg overall, and 104 mmHg, 89 mmHg and 94 mmHg for the smoking, ex-smoking and non-smoking groups respectively. Right foot toe pressures were 98 mmHg overall, and 97 mmHg, 93 mmHg and 106 mmHg for the smoking, ex-smoking and non-smoking groups respectively. It is worth noting that the median toe pressures are lowest for both feet in the ex-smoking group. When assessing left toe pressures respective to ischemia grades, those in the smoking group were significantly more likely to be ischaemic (toe pressure < 30 mmHg) in comparison to the rest of the study population. There were 6 (16.7%) smokers with left toe pressures < 30 mmHg (Fig. 2), versus 5 ex-smokers (6.1%) and 2 never-smokers (2.6%). This was also a trend for the right foot, with 4 smokers (11.1%), compared to 8.5% ex-smokers and 5.2% non-smokers, having grade 3 ischaemia toe pressures, however these results were not significant.

An incidental finding of our data analysis was that macro and microvascular complications were most prevalent in the ex-smoking cohort with 100% of this group having had at least one of neuropathy, retinopathy, nephropathy, peripheral arterial disease, coronary heart disease and/or cerebrovascular disease compared to 87% of the remaining people (p < 0.001). Because neuropathy was present in 96% of ex-smokers, we also analysed the sample complications except neuropathy. Excluding neuropathy, 74% of ex-smokers had one or more of those complications compared to 57% of the other people (*p* = 0.011).

A history of previous minor or major amputation was unexpectedly most prevalent in those who had never smoked (41.6%). The lowest rates of PAD were reported in current smokers (22.2%). Eighty one (41%) participants had healed by end of the follow up period. Fifty-six (28.7%) were not healed at the time they were lost to follow up, most of these were from the non-smoking cohort (36.4%). The highest rate of minor amputations at 12 month follow up was seen in current smokers (8.3%), and least in the non-smokers (2.6%), however this was not statistically significant.

## Discussion

Smoking is a major risk factor for the development and progression of cardiovascular disease and PAD ^30^. There is a substantial effect of smoking on large artery function. Research has reported that tobacco smoking increased the pulse wave velocity suggesting an increase in arterial stiffness ^31^. These investigators also suggested that smoking decreases elasticity in both large and medium sized arteries.

Acute smoking causes increases in both systolic and diastolic blood pressure and heart rate which usually returns to baseline after 15 min ^31^. These effects are also seen in the blood pressure of the aorta following only 1 cigarette in chronic smokers and non-smokers. The greatest changes were seen in the first 5 min after a cigarette. These results suggest that acute smoking has important potential for effects on blood flow to the lower limbs.

In addition, acute smoking causes vasoconstriction which can further decrease blood flow and oxygen delivery ^32,33^. In healthy people, there was a 50% reduction in blood flow in finger circulation, and an over 10% reduction in people with diabetes ^34^. Importantly, this impairment recovers when smoking is ceased ^33^. If there was a 10–50% reduction in toe blood flow with smoking, this would be very likely to impair ulcer healing and increase risk of LLA.

In our study, current smokers had the lowest percentage of PAD out of the three cohorts. However, smokers were also significantly younger which could potentially mean less time for PAD and other macro and microvascular complications to develop. It is also possible that smokers have quit when they were informed that they have PAD or other diabetes complications. Despite having the lowest incidence of complications, the current smokers were also the youngest to ulcerate. It is important to note that our clinic is set up to treat people with diabetes, so the results may be different if the non-diabetic population was greater.

A significant limitation of this study was that brachial pressure was not measured which could have altered results as it is likely those with high blood pressure or calcification of small vessels may have had falsely elevated toe pressures. We were not able to account for time of last cigarette smoked, which as discussed above can potentially have significant acute effects on peripheral blood flow. However, all patients were at least 30 min from being outside of the hospital (where they could potentially smoke) at the time of PPG measurement. Another limitation is that we did not use toe pressures in conjunction with transcutaneous oxygen tension (TcPO2) which can be a more accurate measure in people with diabetes, as smoking can directly influence TcPO2 readings ^13,35^.

Our study confirmed our hypothesis when PPGs were assessed in grades of ischemia, however only for the left leg, with a trend towards more ischaemic toe pressures in the right leg. Our study showed a tendency for higher rates of LLAs in smokers despite their younger age, lower rates of peripheral neuropathy, and fewer previous amputations.

## Conclusion

The link between tobacco smoking and PAD is widely known. This study only demonstrated a small effect of smoking status on systolic toe pressures, however, this was significant when assessed as grades of ischaemic of the left foot. There was an association between age at ulceration in smokers, and a trend towards lower SES and higher minor amputation rates. Future research should be prospective in order to record time of last cigarette smoked, and include brachial measurements for higher accuracy of results.

## Data Availability

The datasets analysed during the current study available from the corresponding author on reasonable request.

## Abbreviations

ABI: Ankle brachial index
BMDH: Blacktown mount druitt hospital
CLI: Critical limb ischaemia
DFU: Diabetic foot ulcer
HRFS: High risk foot service
IRSD: Index of relative socio-economic disadvantage
LLA: Lower limb amputation
PPG: Photoplethysmography
SES: Socioeconomic status
TBI: Toe brachial index
TcPO2: Transcutaneous oxygen tension
